# Risk Factors Associated with Impairment in Pulmonary Diffusing Capacity among Patients with Noncystic Fibrosis Bronchiectasis

**DOI:** 10.1155/2022/8175508

**Published:** 2022-03-09

**Authors:** Kaijun Zhang, Xin Zou, Zhiyi Ma, Xiaohong Liu, Chencheng Qiu, Lingyan Xie, Zhaosheng Lin, Saiyu Li, Yongming Wu

**Affiliations:** ^1^Department of Pulmonary and Critical Care Medicine, Longyan First Affiliated Hospital of Fujian Medical University, Longyan 364000, China; ^2^Department of Respiratory Medicine, Shanghang County Hospital, Longyan 364200, China

## Abstract

This study aims to investigate the risk factors associated with impaired pulmonary diffusing capacity among patients with noncystic fibrosis bronchiectasis (NCFB) and compare the predictive value of several scoring systems for the impairment in these patients. Between July 2019 and June 2021, patients who were admitted to the hospital and diagnosed with NCFB were included in this study. Clinical data were collected and analyzed retrospectively. A total of 175 NCFB patients were included in the analysis. Multivariate logistic regression analysis revealed that impaired pulmonary diffusing capacity diagnosed by carbon monoxide diffusing capacity (DLCO) <80% prediction was associated with age, Reiff score, body mass index (BMI), comorbid chronic obstructive pulmonary disease (COPD), and interstitial lung disease (ILD). Disease duration, frequency of exacerbation, hemoglobin level, and COPD were independent risk factors for impaired pulmonary diffusing capacity diagnosed by DLCO/alveolar volume (VA) <80% prediction. Age, Reiff score, and smoking status were independent risk factors for decreased VA diagnosed by VA <80% prediction. The areas under the curve (AUC) for discrimination of DLCO <80% prediction were 0.822 (0.760–0.885) for Bronchiectasis Severity Index (BSI), 0.787 (0.718–0.856) for FACED, 0.795 (0.729–0.863) for E-FACED, and 0.767 (0.694–0.839) for modified Medical Research Council (mMRC) scores; the AUC for discrimination of DLCO/VA <80% prediction was 0.803 (0.727–0.880) for BSI, 0.752 (0.669–0.835) for FACED, 0.757 (0.676–0.839) for E-FACED, and 0.762 (0.679–0.845) for mMRC, respectively. The BSI had the largest AUC, but the differences between those scoring systems had no statistical significance (*P*=0.181 for DLCO <80% prediction and *P*=0.105 for DLCO/VA <80% prediction). The mMRC score (up to 2 grades) showed a high specificity for discriminating diffusing dysfunction (88.3% for DLCO <80% prediction and 76.1% for DLCO/VA <80% prediction). In NCFB patients, several factors such as age, Reiff score, BMI, exacerbation frequency, disease duration, and comorbid COPD and ILD were associated with impaired pulmonary diffusing capacity, which requires more attention in managing those patients. In addition, several scoring methods, including a simple index of mMRC, showed a comparable and moderate performance for predicting pulmonary diffusing impairment and would facilitate the systematic evaluation of the diffusing capacity of NCFB patients.

## 1. Introduction

Noncystic fibrosis bronchiectasis (NCFB) is a multidimensional disease with various etiologies and multiple mechanisms, leading to different degrees of severity and prognosis [1]. The incidence of NCFB in the UK was 35.2 per 100,000 person-years among women in 2013 and 26.9 per 100,000 person-years among men [2]. Moreover, increasing trends in incidence and mortality have been observed in the UK [2]. Similarly, in Germany, the incidence of NCFB was estimated to be 21.23 per 100,000 inhabitants in 2013 [3]. Among Asians older than 65 years, the prevalence of bronchiectasis is 2.5- to 3.9-fold higher than that in white and black populations [4]. The prevalence of bronchiectasis in Korea was reported to be 9.1% in adults [5]. Although the prevalence of bronchiectasis among the population over 40 years old in China was estimated at 1.2%, the actual prevalence may be higher because only diagnosed patients were included [6].

As a simple, safe, and noninvasive procedure, pulmonary function examination is widely performed for patients with respiratory diseases. Abnormal pulmonary ventilation function is easily found in patients with bronchiectasis, which is associated with the extent and severity of bronchial damage and coexisting factors, such as smoking status and comorbidities such as asthma or chronic obstructive pulmonary disease (COPD). The forced expiratory volume in 1 s (FEV1) is one of the most popular parameters for assessing the degree of lung function impairment in patients with bronchiectasis. It is incorporated into scoring systems for the evaluation of bronchiectasis severity, such as the Bronchiectasis Severity Index (BSI) [7], FACED [8], and E-FACED scores [9]. Moreover, most studies addressing the role of pulmonary function parameters in NCFB patients with lung function impairment focused on pulmonary ventilation parameters, such as FEV1 decline. Other pulmonary functional parameters, such as the pulmonary diffusing capacity, seem to be independent predictors for the mortality of patients with bronchiectasis [10].

Unfortunately, the risk factors associated with impaired pulmonary diffusing capacity in NCFB patients remain unclear. Therefore, additional research is required to identify individuals at high risk of death and improve the management of NCFB. Moreover, the predictive role of the above scoring systems for impaired pulmonary diffusing capacity among NCFB patients remains largely undefined. Thus, another aim of the study is to investigate the predictive roles of the scoring systems.

## 2. Materials and Methods

### 2.1. Ethics

The study protocol was approved by the Ethics Review Committee of the First Hospital of Longyan, Fujian Medical University. Written informed consent was obtained from all patients included in the study.

### 2.2. Subjects

Between July 2019 and June 2021, patients admitted to our center and diagnosed with bronchiectasis using computerized chest tomography (CT) were recruited for further analysis. Patients were excluded if they met the following criteria: (1) cystic fibrosis bronchiectasis, (2) pulmonary tuberculosis, (3) lung cancer, (4) severe immune suppression, such as transplantation and acquired immunodeficiency syndrome (AIDS), (5) new onset of relatively severe pneumonia, and (6) no pulmonary function examination.

### 2.3. Data Collection and Definition

Data, such as age, height, weight, body mass index (BMI), smoking, medical history, symptoms, hemoglobin, acute exacerbation episode, underlying diseases, pulmonary function examinations, and sputum culture, were collected on admission or during hospitalization. Scoring systems, such as the BSI, FACED, E-FACED, modified Medical Research Council (mMRC), and Reiff scores, were estimated and analyzed.

Impaired diffusing capacity was considered as carbon monoxide diffusing capacity (DLCO) or the coefficient of transfer factor (DLCO/alveolar volume (VA)) <80% prediction. Ventilation dysfunction was defined as follows: obstructive ventilation dysfunction (FEV1/forced vital capacity (FVC)% <70% with FEV1% <80% prediction and FVC ≥80% prediction; or FEV1/FVC% <70% with FVC <80% prediction but total lung volume (TLC, %) ≥80% prediction; or FEV1%/FVC% ≥70% with FVC <80% prediction but TLC ≥80% prediction), restrictive ventilation dysfunction (FEV1/FVC% ≥70% with TLC <80% prediction), mixed ventilatory dysfunction (FEV1/FVC% <70% with FVC <80% prediction and TLC <80% prediction), nonspecific ventilation dysfunction (FEV1/FVC ≥70% and TLC >80% prediction, but FEV1 <80% prediction or FVC <80% prediction), air-trapping (residual volume (RV)/TLC% >40%), and overinflation (TLC >120% prediction). The mMRC dyspnea score was evaluated based on the following rules: Grade 0 (I only get breathless with strenuous exercise); Grade 1 (I get short of breath when hurrying on level ground or walking up a slight hill); Grade 2 (I walk slower than people of the same age on level ground because of breathlessness, or I have to stop for breath when walking at my own pace on level ground); Grade 3 (I stop for breath after walking ∼100 meters or after a few minutes on level ground); Grade 4 (I am too breathless to leave the house or I am breathless when dressing); and (3) The modified Reiff score was recorded by assessing the radiographic extension (tubular: 1 point, varicose: 2 points; cystic: 3 points) and the lingula lobe as a separate lobe, with a total score ranging from 0 to 18 points. The BSI [7], FACED [8], and E-FACED [9] scores were all calculated as previously reported.

### 2.4. Statistical Analysis

SPSS 26.0 software was used to perform the statistical analysis. If the normal distribution was observed in the continuous variables, the data were expressed as mean ± standard deviation (SD), and a *t-*test was used to compare the groups. Otherwise, the data were expressed as the median and interquartile range values, and a rank-sum test was used for comparisons between groups. Categorical variables were presented as counts and percentages, and the chi-square test or Fisher's exact test was performed for comparisons. Variables with a *P* value of <0.05 in the univariate analysis were included in the multivariate analysis using stepwise regression models to identify corresponding risk factors. Receiver-operating characteristic (ROC) curves constructed with Stata 15.0 were used to evaluate the predictive value of several measurements or scoring systems for impaired pulmonary diffusing capacity. The sensitivity, specificity, Youden index, area under the curve (AUC), and optimal cutoff values were calculated. A *P* value less than 0.05 was considered to indicate a significant difference.

## 3. Results

### 3.1. Baseline Characteristics

Data on 223 hospitalized patients with NCFB were collected cross-sectionally during the study period. After exclusion, a final cohort of 175 NCFB patients (age range, 16–88 years) was included.  Table 1 shows the characteristics of the included patients. 106 (60.6%) patients were men, and 77 (44%) were smokers or previous smokers. Additionally, 34 (19.4%), 85 (48.6%), and 56 (32.0%) patients were underweight (BMI <18.5 kg/m^2^), normal weight (BMI 18.5∼24 kg/m^2^), and overweight (BMI >24 kg/m^2^), respectively.

**Figure 1:**
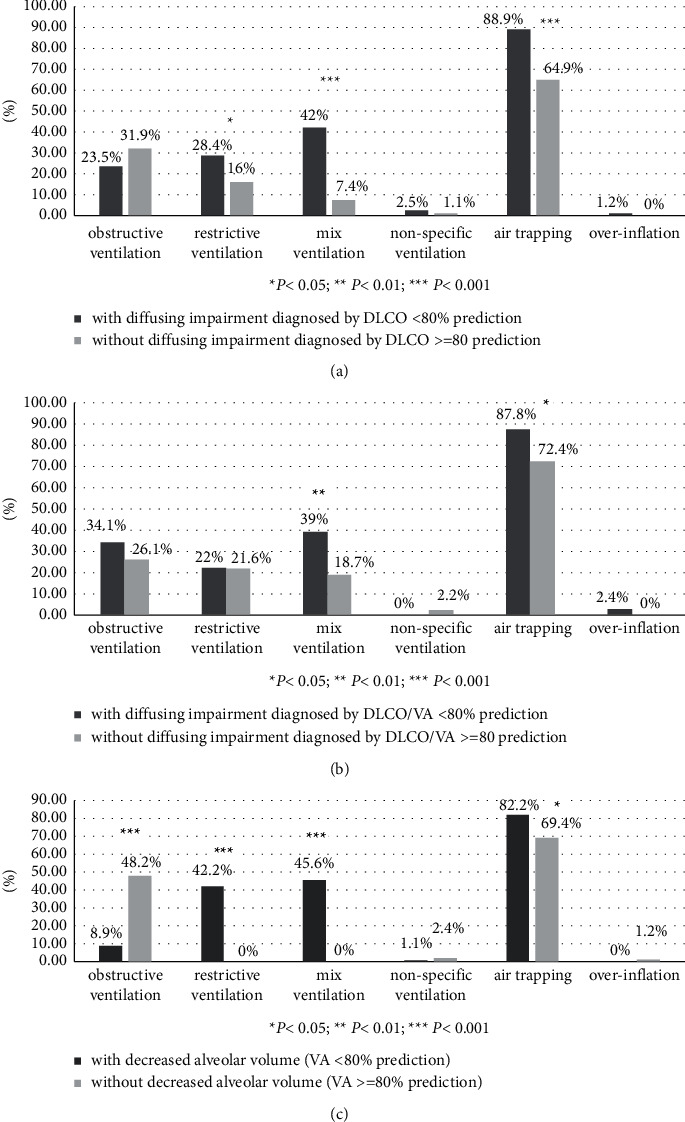
Differences in ventilation pulmonary dysfunction between NCFB patients with and without impaired pulmonary diffusing capacity diagnosed by DLCO <80% prediction (a), with and without impaired pulmonary diffusing capacity diagnosed by DLCO/VA <80% prediction (b), and with and without decreased alveolar volume by VA <80% prediction (c).

Among the 175 patients, 49 (28.0%) had obstructive ventilation dysfunction, 38 (21.7%) had restrictive ventilation dysfunction, 41 (23.4%) had mixed ventilatory dysfunction, 3 (1.7%) had nonspecific ventilation dysfunction, 133 (76.0%) had air trapping, and only one patient had pulmonary overinflation. In addition, 81 (46.3%) patients had diffusion dysfunction diagnosed by DLCO <80% prediction, 41 (23.4%) patients had diffusion dysfunction diagnosed by DLCO/VA <80% prediction, and 90 (51.4%) patients had decreased alveolar volume diagnosed by VA <80% prediction. Patients with restrictive ventilation dysfunction, mixed ventilatory dysfunction, and air-trapping were significantly likely to have pulmonary diffusion dysfunction diagnosed by DLCO <80% prediction (Figure 1(a)). The prevalence of pulmonary diffusion dysfunction diagnosed by DLCO/VA <80% prediction was significantly higher in patients with mixed ventilatory dysfunction and air-trapping (Figure 1(b)). Similarly, decreased VA diagnosed by VA <80% prediction was more likely to happen in patients with restrictive ventilation dysfunction and air-trapping, but less likely to happen in patients with obstructive ventilation dysfunction (Figure 1(c)).

### 3.2. Risk Factors of Impairment in Pulmonary Diffusing Capacity and Decreased Alveolar Volume

Univariate analysis revealed that age, BMI, smoking status, hemoglobin, disease duration, *Pseudomonas aeruginosa* colonization, exacerbation frequency, recent hospitalization (<2 years), Reiff score, cystic bronchial dilation, COPD, asthma, ILD, pulmonary artery hypertension (PAH), and cardiovascular disease were significantly different in patients with and without impaired pulmonary diffuse capacity diagnosed by DLCO <80% prediction. Age, BMI, disease duration, smoking status, hemoglobin, exacerbation and hospitalization frequency (<2 years), Reiff score, COPD, and PAH were significantly different in patients with and without impaired pulmonary diffused capacity diagnosed by DLCO/VA <80% prediction. Age, male sex, BMI, disease duration, smoking status, Reiff score, COPD, and PAH were significantly different in patients with and without VA <80% prediction (Table 1).

Subsequently, multivariate stepwise logistic regression analysis was performed, and the results showed that older age, lower BMI, higher Reiff score, COPD, and ILD were independent risk factors for impaired pulmonary diffusing capacity diagnosed by DLCO <80% prediction among patients with NCFB (Table 2). Disease duration, frequency of exacerbation, hemoglobin, and COPD were independent risk factors for impaired pulmonary diffusing capacity diagnosed by DLCO/VA< 80% prediction (Table 2). Age, Reiff score, and smoking status were independent risk factors for decreased VA diagnosed by VA< 80% prediction (Table 2).

### 3.3. Identification of Impairment in Pulmonary Diffusing Capacity

**Figure 2:**
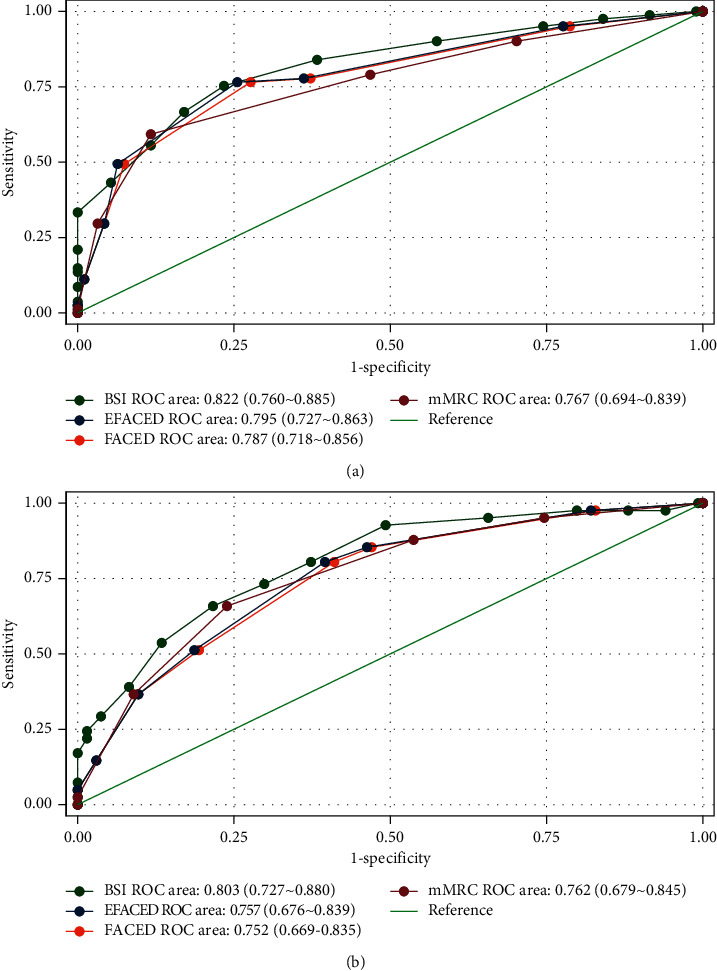
ROC curve analysis of mMRC dyspnea index and different scoring systems for discriminating non-CF bronchiectasis patients with impaired pulmonary diffusing capacity diagnosed by DLCO <80% prediction (a) or DLCO/VA <80% prediction (b).

In our study, the BSI, FACED, E-FACED, and mMRC dyspnea scores were significantly higher in patients with impaired pulmonary diffusion dysfunction (diagnosed by DLCO <80% prediction or DLCO/VA <80% prediction) than in control individuals. The AUCs for the BSI, FACED, E-FACED, and mMRC scores for discriminating those pulmonary function abnormalities were shown (Figures 2(a) and 2(b)). Although the AUC for the BSI score appeared to be the largest, the difference between the BSI score and the others was not statistically significant (*P*=0.181 for DLCO <80% prediction and *P*=0.105 for DLCO/VA <80% prediction).

According to the Youden index, the optimal cutoff values for discriminating DLCO <80% prediction were 11 for the BSI score (sensitivity, 75.2%; specificity, 76.6%; Youden index, 0.519), 3 for the FACED score (sensitivity, 76.5%; specificity, 72.3%; Youden index, 0.488), 5 for the E-FACED score (sensitivity, 76.5%; specificity, 74.5%; Youden index, 0.510), and Grade 2 for the mMRC dyspnea score (sensitivity, 59.3%; specificity, 88.3%; Youden index, 0.476). The optimal cutoff values for discriminating DLCO/VA <80% prediction were 13 for the BSI score (sensitivity, 65.9%; specificity, 78.4%; Youden index, 0.443), 3 for the FACED score (sensitivity, 80.5%; specificity, 59.0%; Youden index, 0.395), 5 for the E-FACED score (sensitivity, 80.5%; specificity, 60.4%; Youden index, 0.409), and Grade 2 for the mMRC dyspnea score (sensitivity, 65.9%; specificity, 76.1%; Youden index, 0.659).

## 4. Discussion

In our study, several factors such as age, BMI, and Reiff score were associated with impaired pulmonary diffusing capacity among NCFB patients. COPD and ILD comorbidities, in particular, had independent effects on pulmonary diffusion impairment (DLCO <80% prediction) in these patients. Further analysis showed that COPD played an independent role in decreasing DLCO/VA. Age and Reiff score played independent roles in decreasing VA. BMI and ILD might show combined effects on pulmonary diffusion through decreased DLCO/VA and VA, although they did not independently affect decreased DLCO/VA and VA when analyzed separately. Except for classical factors such as hemoglobin, disease duration, and frequency of exacerbation would also affect pulmonary diffusion capacity diagnosed by DLCO/VA <80% significantly. However, the BSI scoring system had the most significant area for discrimination of pulmonary diffusing impairment; it just showed a comparable performance compared with several other scoring methods, including a simple index of mMRC. The mMRC index, as a simple tool, would be helpful to evaluate the diffusing capacity of NCFB patients before the initiation of systematic evaluation. Our findings may aid in identifying individuals at high risk of death and improve the management of these patients.

Bronchiectasis is a chronic respiratory disease, and most cases are idiopathic. The typical clinical symptoms include chronic cough, purulent sputum, dyspnea, and hemoptysis. The pathophysiology of the disease is very complex and still poorly understood. Bronchiectasis was previously thought to originate from small airways, gradually leading to the obstruction of more distal airways [11]. Thus, in most literature, bronchiectasis is considered a chronic obstructive airway disease, and an obstructive pattern of lung function impairment is easy to find. Almost all severity scores for bronchiectasis, such as the BSI, FACED, and E-FACED scoring systems, contain only the variable predicted FEV1%. However, a recent study found that in patients with NFCB, pulmonary function abnormality usually presents with air-trapping and diffusion impairment, not airflow obstruction [12]. Besides FEV1, other measures, such as lung diffusing capacity, were independent predictors for mortality in NCFB patients [10]. These recent advances are consistent with our findings. We found that air-trapping is the most common pattern of pulmonary function impairment in NCFB patients, followed by diffusion impairment, obstructive dysfunction, mixed ventilatory dysfunction, restrictive ventilation dysfunction, nonspecific dysfunction, and overinflation.

Our data suggested that the impairment of pulmonary diffusing capacity is independently correlated with age, Reiff score, BMI, COPD, and ILD. Previously, similar findings have been reported by others, and the data also supported our conclusion. For example, an accelerated decline in lung diffusion function was correlated with the length of the disease, and a significant relationship was identified between DLCO decline and age and FEV1 decline [13]. In our study, patients with an increase in the modified Reiff score by 1 point had a 1.23-fold increased risk of pulmonary diffusion impairment. This may be because the modified Reiff score includes bronchodilation degree (cystic, varicose, or column) and the number of lobes involved [14–16]. The association between BMI and the impairment of pulmonary diffusing capacity may be explained by a significant correlation between the basic nutritional status and bronchiectasis development [17, 18]. Moreover, patients with COPD had a 3.296-fold higher odds of developing lung diffusion impairment than those without COPD. This phenomenon is closely related to the association between underlying conditions (such as COPD and asthma) and poor outcomes in NCFB patients, for example, mortality, exacerbation frequency, airway obstruction, pathogen microorganism isolation, and worse quality of life [12, 19]. It is worth noting that although coexistence with asthma was found to be associated with mortality, we did not observe any effects on the pulmonary diffusing capacity in NCFB patients. This may be because asthma is a small-airway disease, and it affects the prognosis of the disease in other aspects, such as frequent exacerbation or worse obstructive ventilation.

In our study, although univariate analysis revealed that *Pseudomonas aeruginosa* colonization is associated with impaired pulmonary diffusing capacity, the association was not confirmed in multivariate regression analysis. Similar findings were reported by King et al. [13] and Guan et al. [15]. However, it remains controversial whether *Pseudomonas aeruginosa* colonization directly impacts lung function. In China, a retrospective multicenter study showed that *Pseudomonas aeruginosa* colonization could easily lead to abnormalities in pulmonary ventilation and diffusion function, which may further result in unfavorable outcomes, such as dyspnea acute exacerbation severe anxiety and depression, and even mortality. Besides these, *Pseudomonas aeruginosa* colonization is also associated with a significant annual decline in FEV1 [20]. Therefore, it is easy to conclude that *Pseudomonas aeruginosa* colonization could be used to indicate disease severity. Further study is required to investigate the association between *Pseudomonas aeruginosa* colonization and pulmonary diffusion function in the future.

BSI, FACED, and E-FACED are three scoring systems recommended to evaluate bronchiectasis severity. The three scoring systems all constitute the predicted FEV1% variable, which represents the degree of obstruction. In our study, the BSI scoring system appeared to have the largest AUC in predicting impaired pulmonary diffusing capacity among NCFB patients. However, the difference between BSI and other systems did not reach statistical significance. For comparing the clinical utility of those scoring systems, many validated cohorts were examined. The results demonstrated that the BSI score outperformed other systems for predicting the decline of activity tolerance and lung function, including the risk of acute exacerbation and hospitalization [20], but was comparable for predicting mortality [21, 22].

In addition, unlike multidimensional scoring systems, the mMRC scoring system is straightforward to use. The index was mainly designed for the clinical evaluation of activity tolerance. In our study, the predictive capacity of the mMRC scoring system was moderate for discriminating between patients with and without lung diffusing dysfunction. Although the AUC of mMRC appeared to be the smallest, the system showed high specificity (88.3% for DLCO <80% prediction and 76.1% for DLCO/VA <80% prediction). Therefore, the mMRC scoring system is a helpful screening tool for the initial evaluation of lung function, even in cost-constrained healthcare environments.

## 5. Conclusion

In NCFB patients, age, low BMI, High Reiff score, and comorbidity with COPD and ILD are independent risk factors for impaired pulmonary diffusing capacity diagnosed by DLCO <80% prediction. Besides hemoglobin, frequency of exacerbation and disease duration also significantly affect pulmonary diffusion (DLCO/VA <80% prediction). In addition, our study suggests that the BSI, FACED, and E-FACED scoring systems show desirable predictive ability for impaired pulmonary diffusing capacity in NCFB patients; and that mMRC, as a simple screening tool, is useful even in cost-constrained healthcare environments.

## Figures and Tables

**Table 1:** Clinical characteristics of NCFB patients with and without impaired pulmonary diffusing capacity (DLCO <80% prediction and DLCO/VA <80% prediction) and decreased alveolar volume (VA <80% prediction).

	Total (*n*)	Impaired diffusing capacity diagnosed by DLCO		*P* value	Impaired diffusing capacity diagnosed by DLCO/VA		*P* value	Decreased VA		*P* value
		≥80% prediction	<80% prediction		≥80% prediction	<80% prediction		≥80% prediction	<80% prediction	
Number	175	94	81		134	41		85	90	
Age (years)	175 (100%)	58.17 ± 12.53	66.84 ± 11.68	<0.001	60.66 ± 12.59	67.17 ± 12.61	0.004	58.62 ± 13.43	65.54 ± 11.39	<0.001
Sex, male	106 (60.5%)	53 (56.4%)	53 (65.4%)	0.22	77 (57.5%)	29 (70.7%)	0.128	41 (48.2%)	65 (72.2%)	0.001
Hemoglobin (g/L)	175 (100%)	133.17 ± 17.36	125.99 ± 16.84	0.006	131.29 ± 12.60	135.10 ± 12.61	0.047	129.98 ± 17.12	129.69.10 ± 17.83	0.914
Duration of disease (years)	175 (100%)	0.84 (0.00, 5.00)	6.00 (1.75, 10.00)	<0.001	2.00 (0, 6.00)	10.00 (5.00, 12.50)	<0.001	2.00 (0, 7.00)	5.00 (0, 10.00)	0.006
Modified Reiff score	175 (100%)	5.00 (3.00, 8.00)	8.00 (5.00, 12.00)	<0.001	6.00 (4.00, 9.25)	8.00 (4.00, 12.00)	0.037	5.00 (3.00, 8.00)	8.00 (5.00, 11.00)	<0.001
Frequency of hospitalization in the last 2 years	175 (100%)	1.00 (1.00, 1.00)	1.00 (1.00, 2.00)	0.02	1.00 (1.00, 1.00)	2.00 (1.00, 2.00)	<0.001	1.00 (1.00, 1.00)	2.00 (1.00, 2.00)	0.108
Frequency of exacerbation in the last 2 years	175 (100%)	1.00 (1.00, 2.00)	2.00 (1.00, 3.00)	<0.001	1.00 (1.00, 2.00)	3.00 (1.50, 3.00)	<0.001	1.00 (1.00, 2.00)	2.00 (1.00, 3.00)	0.083
Body mass index (kg/m^2^)										
<18.5	34 (19.4%)	9 (9.6%)	25 (30.9%)	<0.001	19 (14.2%)	15 (36.6%)	0.003	11 (12.9%)	23 (25.6%)	0.031
18.5∼24	85 (48.6%)	43 (45.7%)	42 (51.9%)		66 (49.3%)	19 (46.3%)		40 (47.1%)	45 (50.0%)	
≥24	56 (32.0%)	42 (44.7%)	14 (17.3%)		49 (36.6%)	7 (17.1%)		34 (40.0%)	22 (24.4%)	
Smoking status										
Smoker or ex-smoker	77 (44.0%)	32 (34.0%)	45 (55.6%)	0.005	53 (39.6%)	24 (58.5%)	0.032	28 (32.9%)	49 (54.4%)	0.004
Nonsmoker	98 (56.0%)	62 (66.0%)	37 (44.4%)		81 (60.4%)	17 (41.5%)		57 (67.1%)	41 (45.6%)	
*Pseudomonas aeruginosa* colonization										
Yes	12 (6.9%)	3 (3.2%)	9 (11.1%)	0.04	8 (6.0%)	4 (9.8%)	0.627	4 (4.7%)	8 (8.9%)	0.274
No	163 (93.2%)	91 (96.8%)	72 (88.9%)		126 (94.0%)	37 (90.2%)		81 (95.3%)	82 (91, 1%)	
Lower airway pathogen detection										
None	122 (69.7%)	72 (76.6%)	50 (61.7%)	0.08	97 (72.4%)	25 (61.0%)	0.212	64 (75.3%)	58 (64.4%)	0.187
*Pseudomonas aeruginosa*	22 (12.61%)	8 (8.5%)	14 (17.3%)		17 (12.7%)	5 (12.2%)		7 (8.2%)	15 (16.7%)	
Other pathogen	31 (18.9%)	14 (14.9%)	17 (21.0%)		20 (14.9%)	11 (26.8%)		14 (16.5%)	17 (18.9%)	
Chronic obstructive pulmonary disease										
Yes	64 (36.6%)	20 (21.3%)	44 (54.3%)	<0.001	35 (26.1%)	29 (70.7%)	<0.001	23 (27.1%)	41 (45.6%)	0.011
No	111 (63.4%)	74 (78.7%)	37 (45.7%)		99 (73.9%)	12 (29.3%)		62 (72.9%)	49 (54.4%)	
Asthma										
Yes	20 (11.4%)	16 (17.0%)	4 (4.9%)	0.01	18 (13.4%)	2 (4.9%)	0.220	13 (15.3%)	7 (7.8%)	0.118
No	155 (88.6%)	78 (83.0%)	77 (95.1%)		116 (86.6%)	39 (95.1%)		72 (84.7%)	83 (92.2%)	
Interstitial lung disease										
Yes	13 (7.4%)	1 (7.7%)	12 (92.3%)	0.001	8 (6.0%)	5 (12.2%)	0.322	4 (4.7%)	9 (10.0%)	0.182
No	162 (92.6%)	93 (57.4%)	69 (42.6%)		126 (94.0%)	36 (87.8%)		81 (95.3%)	81 (90.0%)	
Pulmonary arterial hypertension										
Yes	27 (15.4%)	4 (4.3%)	23 (28.4%)	0.001	8 (6.0%)	5 (12.2%)	<0.001	8 (9.4%)	19 (21.1%)	0.032
No	148 (84.6%)	90 (95.7%)	58 (71.6%)		126 (94.0%)	36 (87.8%)		77 (90.6%)	71 (78.9%)	
Cardiovascular diseases										
Yes	56 (32.0%)	23 (25.3%)	33 (40.7%)	0.02	38 (28.4%)	18 (43.9%)	0.062	25 (29.4%)	31 (34.4%)	0.476
No	119 (68.0%)	71 (74.7%)	48 (59.3%)		96 (71.6%)	23 (56.1%)		60 (70.6%)	59 (65.6%)	
Diabetes mellitus										
Yes	20 (11.4%)	12 (12.8%)	8 (9.9%)	0.55	16 (11.9%)	4 (9.8%)	0.917	11 (12.9%)	9 (10.0%)	0.541
No	155 (88.6%)	82 (87.2%)	73 (90.1%)		118 (88.1%)	37 (90.2%)		74 (87.1%)	81 (90.0%)	
Gastroesophageal reflux disease										
Yes	21 (12.0%)	12 (12.8%)	9 (11.1%)	0.74	17 (12.7%)	4 (9.8%)	0.818	7 (8.2%)	14 (15.6%)	0.136
No	154 (88.0%)	82 (87.2%)	72 (88.9%)		117 (87.3%)	37 (90.2%)		78 (91.8%)	76 (84.4%)	
Other complications										
Yes	85 (48.6%)	48 (51.1%)	37 (45.7%)	0.48	65 (48.5%)	20 (48.8%)	0.976	41 (48.2%)	44 (48.9%)	0.931
No	90 (51.4%)	46 (48.9%)	44 (54.3%)		69 (51.5%)	21 (51.2%)		44 (51.8%)	46 (51.1%)	
Bronchi cystic dilation										
Yes	92 (52.6%)	43 (45.7%)	49 (60.5%)	0.05	67 (50.0%)	25 (61.0%)	0.218	40 (47.1%)	52 (57.8%)	0.156
No	83 (47.4%)	51 (54.3%)	32 (39.5%)		67 (50.0%)	16 (39.0%)		45 (52.9%)	38 (42.2%)	
Bronchi varicose dilation										
Yes	110 (62.9%)	55 (58.5%)	55 (67.9%)	0.20	83 (61.9%)	27 (65.9%)	0.650	52 (61.2%)	58 (64.4%)	0.655
No	65 (37.1%)	39 (41.5%)	26 (32.1%)		51 (38.1%)	14 (34.1%)		33 (38.8%)	32 (35.6%)	
Bronchi column dilation										
Yes	122 (69.7%)	65 (69.1%)	57 (70.4%)	0.86	92 (68.7%)	30 (73.2%)	0.582	59 (69.4%)	63 (70.0%)	0.933
No	53 (30.3%)	29 (30.9%)	25 (29.7%)		42 (31.3%)	11 (26.8%)		26 (30.6%)	27 (30.0%)	

**Table 2:** Multivariate analysis of risk factors associated with impaired pulmonary diffusing capacity diagnosed by DLCO <80% prediction (a) and DLCO/VA <80% prediction (b), and decreased alveolar volume diagnosed by VA<80% prediction (c) among non-CF bronchiectasis patients.

Age (years)	*(a) Multivariate analysis of impaired pulmonary diffusing capacity diagnosed by DLCO <80% prediction*					
	Β-value	SE value	Wald value	OR value	95% CI	*P* value

	0.475	0.019	6.346	1.048	1.010∼1.087	0.0
Body mass index						
<18.5	1.250	0.525	5.667	3.490	1.247∼9.766	0.017
≥24	−0.855	0.461	3.437	0.425	0.172∼1.050	0.064
18.5∼24						
Chronic obstructive pulmonary disease	1.217	0.413	8.693	3.377	1.504∼7.582	0.003
				18.31	2.959∼171.2	
Interstitial lung disease	2.908	1.114	6.501	6	32	0.011
Modified Reiff score	0.202	0.057	12.577	1.224	1.094∼1.368	<0.001

*(b) Multivariate analysis of impaired pulmonary diffusing capacity diagnosed by DLCO/VA <80% prediction*						
	Β-value	SE value	Wald value	OR value	95% CI	*P* value
Duration of disease (years)	0.061	0.020	9.866	1.063	1.023∼1.105	0.002
Frequency of exacerbation in the last 2 years	0.200	0.108	3.440	1.221	0.989–1.508	0.064
Hemoglobin (g/L)	−0.033	0.013	6.077	0.968	0.943∼0.993	0.014
					3.178∼18.50	
Chronic obstructive pulmonary disease	2.037	0.449	20.547	7.669	5	<0.001

*(c) Multivariate analysis of decreased alveolar volume diagnosed by VA <80% prediction*						
	Β-value	SE value	Wald value	OR value	95% CI	*P* value
Age (years)	0.037	0.019	6.124	1.038	1.008∼1.069	0.013
Modified Reiff score	0.238	0.051	21.674	1.269	1.148∼1.408	<0.001
Smokers or ex-smokers	1.004	0.367	7.490	2.729	1.330∼5.602	0.006

## Data Availability

The data used to support the findings of this study are available from the corresponding author upon request.

## Abbreviations

NCFB: Noncystic fibrosis bronchiectasis
COPD: Chronic obstructive pulmonary disease
ILD: Interstitial lung disease
PAH: Pulmonary artery hypertension
BMI: Body mass index
BSI: Bronchiectasis Severity Index
mMRC: Modified Medical Research Council
CT: Computerized tomography
AIDS: Acquired immunodeficiency syndrome
DLCO: Carbon monoxide diffusing capacity
VA: Alveolar volume
DLCO/VA: Carbon monoxide diffusing capacity divided by alveolar volume
FEV1: Forced expiratory volume in 1 s
FVC: Forced vital capacity
TLC: Total lung volume
RV: Residual volume
SD: Standard deviation
ROC: Receiver-operating characteristic
AUC: Area under the curve.
